# Supplementary figures and images for: Sparse deep predictive coding captures contour integration capabilities of the early visual system
Source: PLoS Comput Biol. 2021 Jan 26;17(1):e1008629. doi: 10.1371/journal.pcbi.1008629 (PMC7864399; doi:10.1371/journal.pcbi.1008629)

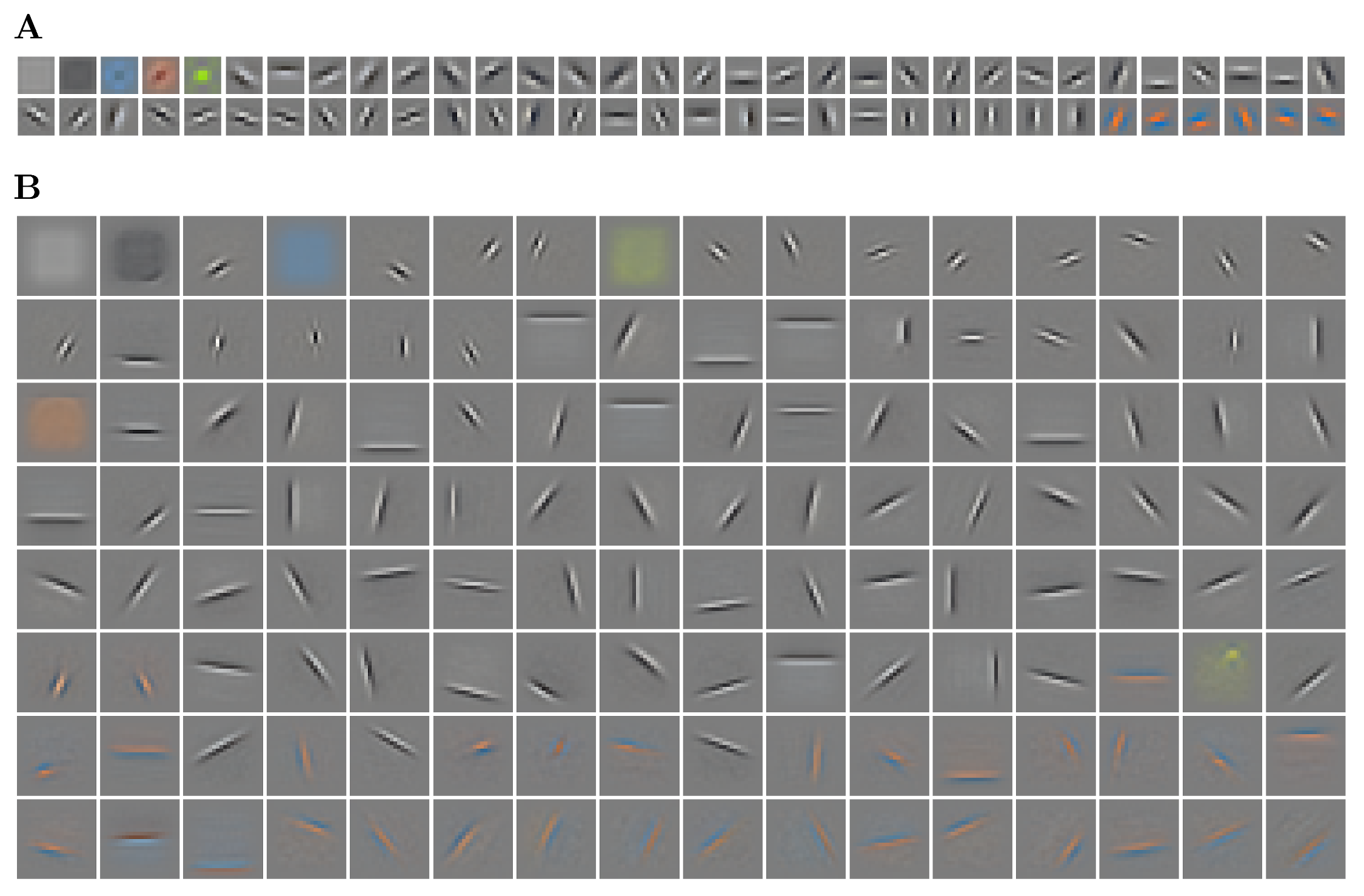

Supplement: S1 Fig — (A) 64 first layer RFs, sorted by activation probability in a descending order. The size of the RFs is 9 × 9 px. (B) 128 second layer RFs, sorted by activation probability in a descending order. The size of the RFs is 22 × 22 px. All the visualized RFs are generated using Eq 3. (TIF) [file pcbi.1008629.s001.tif]

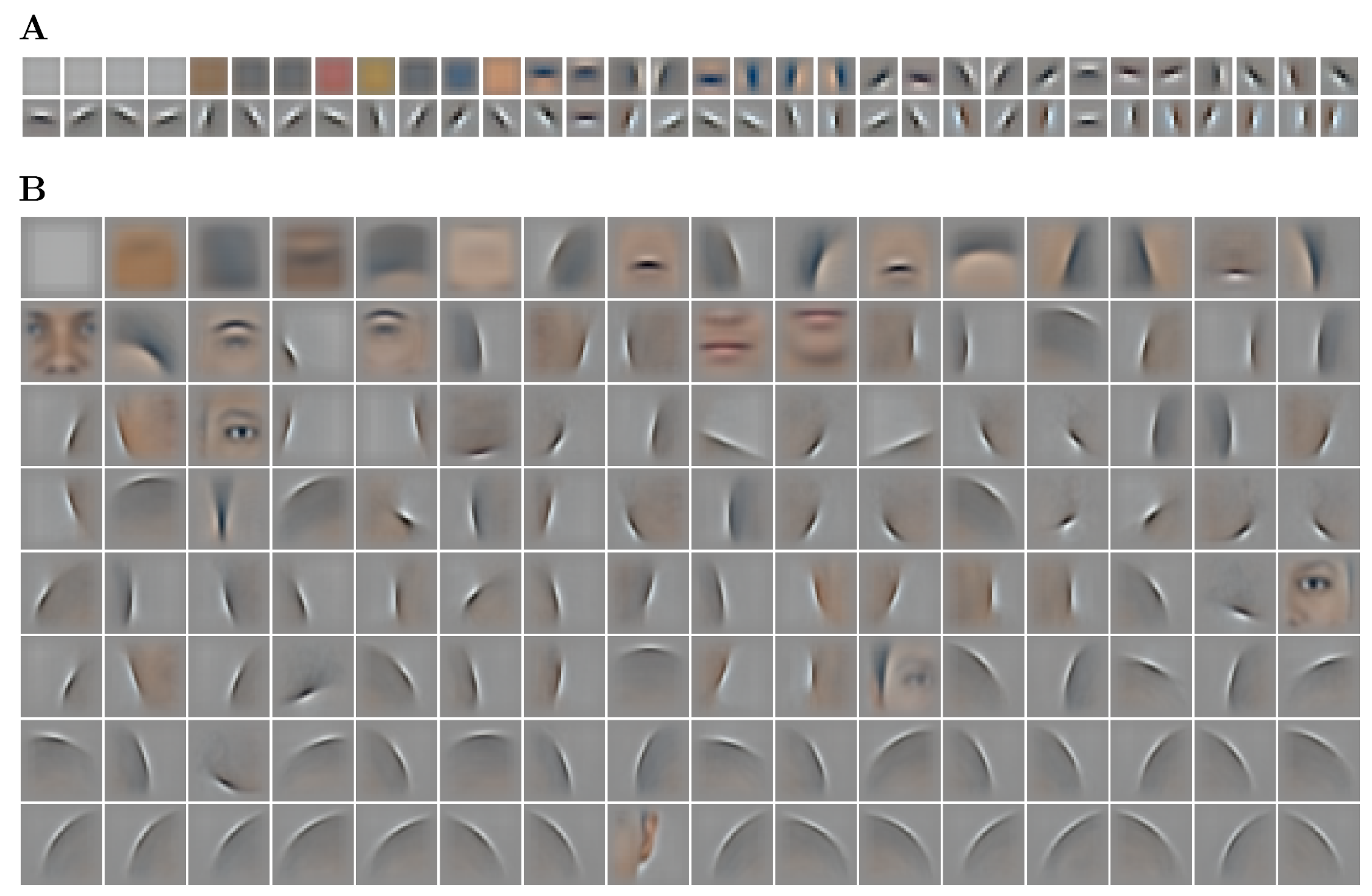

Supplement: S2 Fig — (A) 64 first layer RFs, sorted by activation probability in a descending order. The size of the RFs is 9 × 9 px. (B) 128 second layer RFs, sorted by activation probability in a descending order. The size of the RFs is 33 × 33 px. All the visualized RFs are generated using Eq 3. (TIF) [file pcbi.1008629.s002.tif]

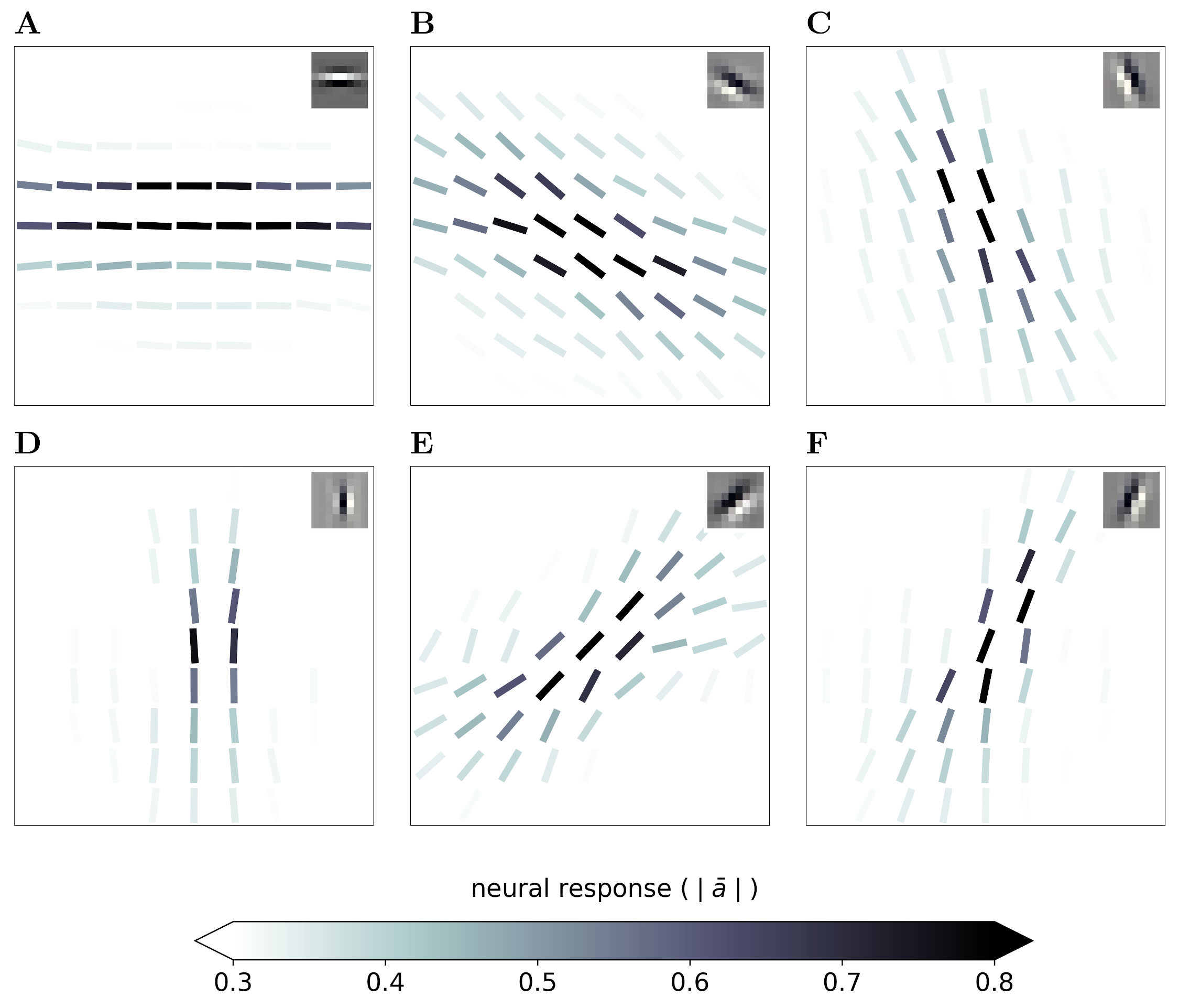

Supplement: S3 Fig — From left to right and top to bottom the contour orientations are 0° (A), −30° (B), −60° (C), 90° (D), 60° (E) and 30° (F). The feedback strength is set to 1. At each location identified by the coordinates (xc, yc) the angle is θ¯[xc,yc] (see Eq 11) and the color scale is |a¯[xc,yc]| (see Eq 12). The color scale being saturated toward both maximum and minimum activity, all the activities above 0.8 or below 0.3 have the same dark green or white color, respectively. (TIF) [file pcbi.1008629.s003.tif]

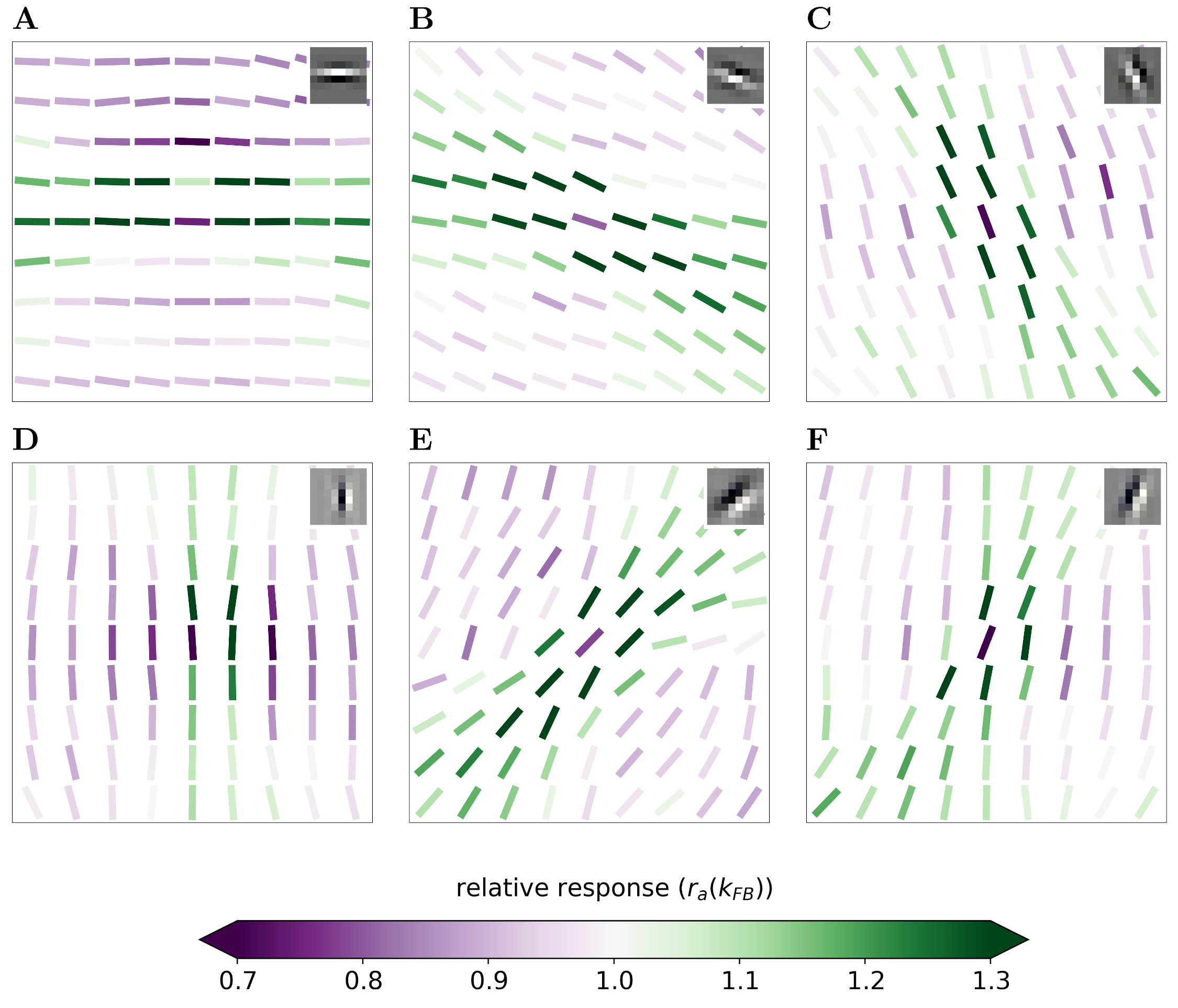

Supplement: S4 Fig — The feedback strength is set to 1. From left to right and top to bottom the contour orientations are 0° (A), −30° (B), −60° (C), 90° (D), 60° (E) and 30° (F). At each location identified by the coordinates (xc, yc) the angle is θ¯[xc,yc] (see Eq 11) and the color scale is proportional to ra(kFB) (see Eq 25). The color scale being saturated toward both maximum and minimum activity, all the activities above 1.3 or below 0.5 have the same dark green or dark purple color, respectively. (TIF) [file pcbi.1008629.s004.tif]

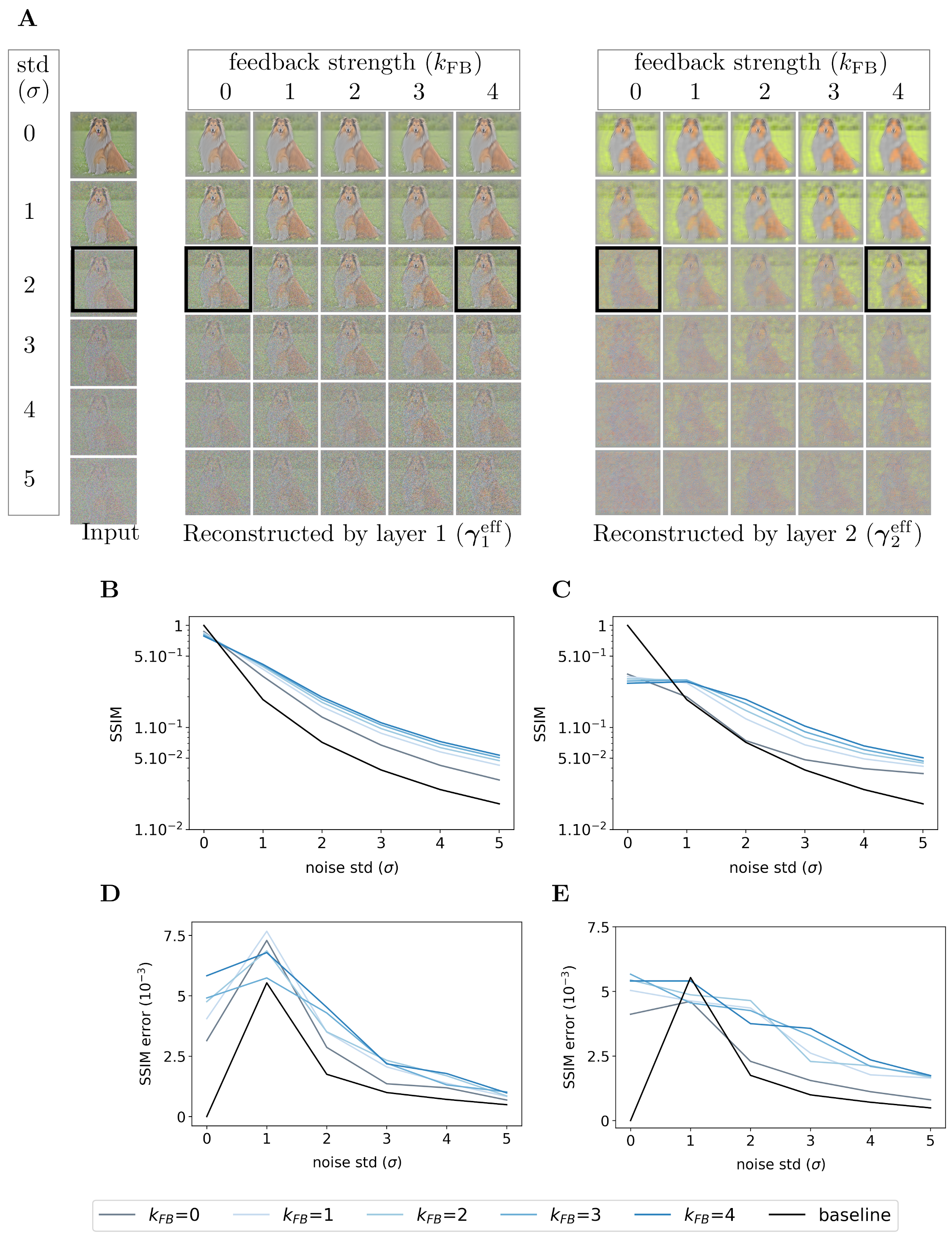

Supplement: S5 Fig — (A) In the left block, one image is corrupted by Gaussian noise of mean 0 and standard deviation (σ) varying from 0 to 5. The central block exhibits the representations made by the first layer (γ1eff), and the right-hand block the representations made by the second layer (γ2eff). Within each of these blocks, the feedback strength (kFB) is ranging from 0 to 4 in columns. Highlighted images with black square are those selected in Fig 10. (B) median structural similarity index between 1200 original images and their reconstructions by the first layer of the SDPC. (C) Structural similarity index between original images and their reconstructions by the second layer of the SDPC. (D) Error, as computed with the median absolute deviation, of the structural similarity index plotted in (B) (i.e. for the first layer). (E) Error, as computed with the median absolute deviation, of the similarity index plotted in (C) (i.e. for the second layer). The color code corresponds to the feedback strength, from light grey for kFB = 0 to darker blue for higher feedback strength. The black line is the baseline, it is the similarity between noisy and original input image. (TIF) [file pcbi.1008629.s005.tif]

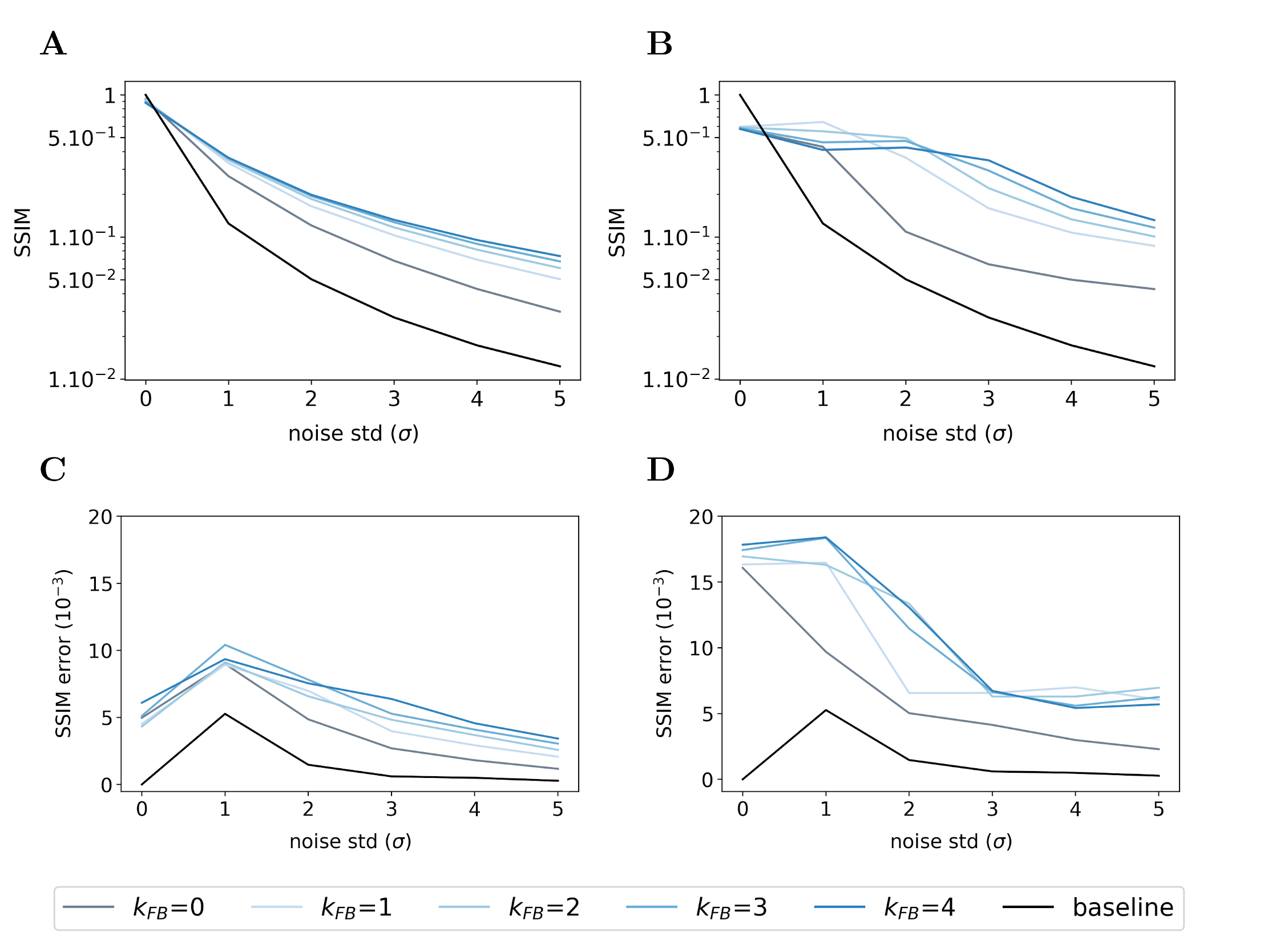

Supplement: S6 Fig — This figure description is similar to the description of the S5 Fig. For this database, all presented curves represent the median structural similarity index over 400 samples of the testing set. (TIF) [file pcbi.1008629.s006.tif]

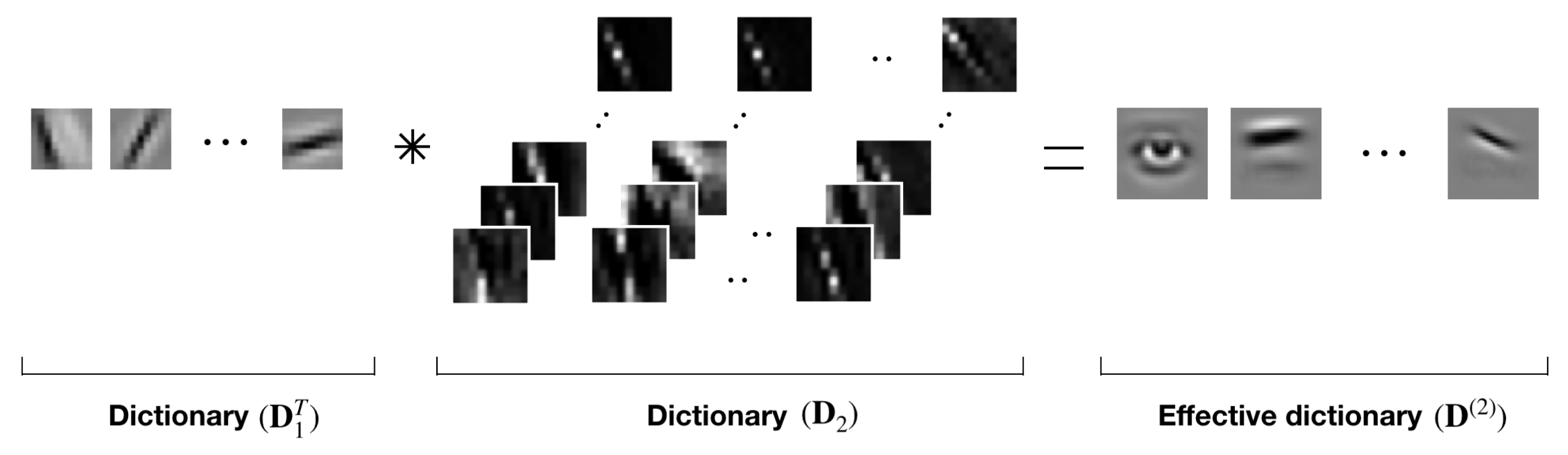

Supplement: S7 Fig — The projection of the second layer dictionary into the visual space (D2effT) is obtained by convolving the transpose of the first layer dictionary (D1T) by the second layer dictionary (D2T) [37]. This mechanism could be also used to back-project any activity map into the visual space (see Eq 18). (TIF) [file pcbi.1008629.s007.tif]

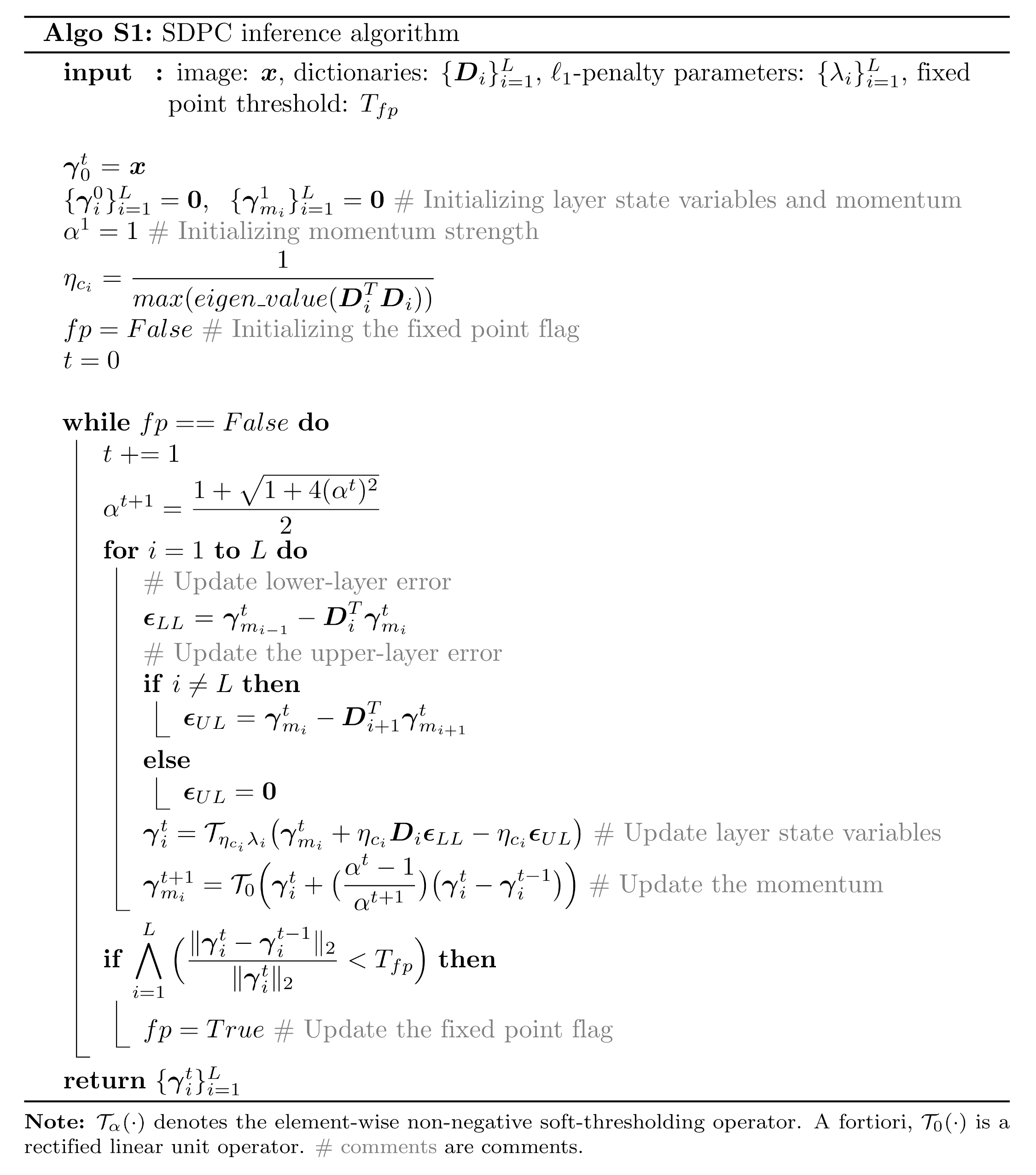

Supplement: S1 Algo — Pseudo-code of the inference using python-like pseudo algorithm. Tα(·) denotes the element-wise non-negative soft-thresholding operator. A fortiori, T0(·) is a rectified linear unit operator. # comments are comments. (TIF) [file pcbi.1008629.s008.tif]
