# Supplementary material for: Sparse deep predictive coding captures contour integration capabilities of the early visual system
Source: PLoS Comput Biol. 2021 Jan 26;17(1):e1008629. doi: 10.1371/journal.pcbi.1008629 (PMC7864399; doi:10.1371/journal.pcbi.1008629)
Supplement: Attachment — Submitted filename: Response_to_reviewer.pdf [file pcbi.1008629.s009.pdf]

April 20, 2020

Victor Boutin
Institute of Neuroscience of la Timone
27, boulevard Jean Moulin
13005 Marseille, France
Email: victor.boutin@univ-amu.fr

Dear editor,

Thank you for allowing us to re-submit an updated version of the paper entitled "*Sparse Deep Predictive Coding captures contour integration capabilities of the early visual system*". We wish to thank both reviewers, because their remarks have allowed us to greatly improve the manuscript by clarifying the text and add new results. The enclosed manuscript has been thoroughly amended to clarify the misunderstanding with the reviewer #1 and to answer the concerns raised by the reviewer #2. Please find below a point-by-point description of the changes we have made in the manuscript :

- **Differences between Auto-Encoders (AEs) and the Sparse Deep Predictive Coding (SPDC) model.** The reviewer #1 erroneously considered that the proposed model was an AEs. While the SDPC aims at reconstructing the previous layer representation, AEs reconstruct the input image. This specific point implies a major difference in the way both frameworks are trained: AEs are trained with back-propagation on a global loss function while the SDPC is trained to jointly minimize several local loss functions (one loss per layer). To dissipate any further misunderstandings, we have now explicitly listed the differences between both models in the Introduction (in paragraph §3) and we have reminded what is making the SDPC unique compared to AEs in the Discussion (subsection: "*SDPC learns cortex-like RFs while performing neuro-plausible computation*").
The reviewer #1 also suggested that the denoising ability of the SDPC was similar to the one of a Denoising AEs. While this is an emergent property of the SDPC (i.e. our model is trained using only non-degraded images), AEs are, on the contrary, explicitly trained to denoise degraded input images. To highlight this crucial difference, we have reformulate the section "*Effect of the feedback at the representational level*" (subsection "*Denoising abilities from the feedback signals of the SDPC*"). In addition, we have further discussed this difference in the Discussion (in subsection: "*SDPC accounts for object processing in V1 with degraded images*).
- **Clarification of the SDPC loss function and the feedback strength**
The reviewer #2 asked for clarifications concerning the SDPC loss function and the feedback strength scalar (denoted k_{FB}). As the SDPC is jointly minimizing one loss function for each layer, we cannot express the mathematical optimisation problem using a unique global loss function. To make this crucial point clearer we have detailed the set of loss functions for a 2-layers SDPC network in the section "*Brief description of the SDPC*". This new mathematical formulation emphasizes the fact that the scalar modeling the feedback strength (ie k_{FB}) is included only in the loss function of the first layer. Therefore, k_{FB} penalizes the reconstruction error with the input image in favor of a better reconstruction error with the 2nd layer representation. In addition, we made clearer the fact that the scalar k_{FB} could be used to assess the effect of interconnecting layer using the Predictive Coding theory. Indeed, when $k_{\mathrm{FB}} = 0$, the SDPC is equivalent to the stacking of independent LASSO sub-problems

(see section "*Brief description of the SDPC*").
As correctly raised by reviewer #2, the word "disambiguation" refers to a better recognition accuracy. We have dissipated the underlying misunderstanding by replacing it by the term "better reconstruction". We thank the reviewer #2 for pointing out this important details to our attention.

- **Better emphasis on the effect Predictive Coding**
The reviewer #2 was concerned about the small effect of Predictive Coding in the presented results. To answer this point we have included 2 different modifications. First, we have modified our analysis in section "*Effect of the feedback at the neural level*" (see Fig 5 in subsection "*SDPC signals reorganize the interaction map of the V1 model*"). Instead of reporting the absolute co-linearity and co-circularity in the interaction maps, we have plotted their relative change when the feedback connection was activated. This new analysis reinforces the message that the feedback tends to modify neural selectivity toward co-linearity in both the end-zone and the side-zone. Second, in section "*Effect of the feedback at the representational level*" (subsection "*Denoising abilities emerge from the feedback signals of the SDPC*"), we have changed the Fig 9. and Fig 10. to better zoom in the qualitative effect of the feedback connection. The previous figures, more complete but less clear, have been appended as Supplementary Informations (see Fig S5. and Fig S6.).

- **New analysis to assess the effect of sparse coding**
The reviewer #2 correctly pointed out that we did not investigate the effect of Sparse Coding in denoising degraded images. Therefore, we have added a new subsection (subsection "*Effect of sparsity on denoising*"), including 2 new figures (Fig 11. and Fig 12.) to address the suggestion of Reviewer #2. This analysis reveals that sparsity is playing a significant role in reconstructing degraded images. In particular, we observed that high sparsity was optimal to reconstruct strongly degraded input while lower sparsity gave better results in reconstructing clearer images. We discuss this result in subsection "*SDPC accounts for object processing in V1 with degraded images*", and concluded that similarly to the feedback strength, sparsity might be subject to attentional modulations. We wish to thank the reviewer 2 for this suggestion.

- **Emphasize on the SDPC novelties compared to the original Rao & Ballard model**
The reviewer #2 mentioned that the presented model was similar to the original Predictive Coding model (from Rao & Ballard) and was concerned about the lack of novelties of the SDPC. We fully agree that the proposed model is inspired from the work of Rao & Ballard, but the SDPC is showing 2 major advancements. First we scale the neural network from dozen of neurons looking at 16×16 imagelets (Rao & Ballard) to a scale which is more realistic for visual processing (hundreds of thousands of neurons looking at higher-resolution images). Second, as the SDPC is a convolutional neural network (versus patch-based for Rao & Ballard), we were able to analyze the neural selectivity and activity outside of the classical RFs. This extension led us to the concept of interaction map. To highlight these differences, we have added a subsection in the Discussion (subsection "*SDPC extends Rao & Ballard's PC model*") to detail all the extensions proposed by the SDPC. In addition, we have explicitly defined the classical and extra-classical RFs in the presented interaction maps (see Fig 4. and Fig 6.) to better visualize the spatial extent of our analysis.

Please find attached to this letter the new manuscript as well as the changes from the previously submitted version in supplementary information (i.e. tracked-changes format). We believe that the modifications brought to the manuscript are responding to the insightful comments of Reviewer #2 while disambiguating the paper to avoid the misunderstanding of reviewer #1. We are convinced that our paper would fit in your journal as it is an extremely timely proposal of better integrating neurophysiological knowledge in current "deep networks".

Sincerely yours,

The authors, Victor Boutin, Angelo Franciosini, Frederic Chavane, Franck Ruffier and Laurent Perrinet
