# Supplementary material for: Sparse deep predictive coding captures contour integration capabilities of the early visual system
Source: PLoS Comput Biol. 2021 Jan 26;17(1):e1008629. doi: 10.1371/journal.pcbi.1008629 (PMC7864399; doi:10.1371/journal.pcbi.1008629)
Supplement: Attachment — Submitted filename: ResponseToReviewers.pdf [file pcbi.1008629.s010.pdf]

October 13, 2020

Victor Boutin
Institute of Neuroscience of la Timone
27, boulevard Jean Moulin
13005 Marseille, France
Email: victor.boutin@univ-amu.fr

Dear reviewers,

We wish to thank both reviewers, because their remarks have allowed us to greatly improve the clarity of the manuscript. The enclosed manuscript has been thoroughly amended to answer the comments made by the reviewers. We would like to draw the reviewer's attention to the fact that all the images used in the manuscript has been changed to fulfill the copyright requirement of the journal. Therefore, all face images has been removed, and all other images has been replaced with images extracted from an open-source database. Note that all line references in this rebuttal letter correspond to line numbers in the tracked changes version of the article. Please find below a point-by-point description of the changes we have made in the manuscript:

Response to reviewer #3:

- **1. Reviewer #3 suggested to include more comparison with experimental data observed in neurophysiology**. Neurophysiological datas being highly specific to the recording methods, the models (monkeys, cats...) and the experimental protocol (types of stimuli, degree of attentional cues...), it is difficult to directly quantitatively compare our results with neural data. Nevertheless, we agree with reviewer #3 that such comparison would be valuable. We thus have included the qualitative trends observed in neurophysiology in our Fig. 7 and Fig. 8 (we have added black arrows in the figures to represent the trends). In addition, we have added a paragraph to thoroughly explain the trend similarities between biological datas and our SDPC model in the section "*Discussion*" (see lines 537-559).
- **2. Reviewer #3 suggested to better define the biological question addressed by the paper**. We fully agree with Reviewer #3 that the biological question raised by our study needs to be better highlighted. To do so, we have updated the paragraph § 5 of the section "*Introduction*" (see lines 82-85). Our biological question is to study the effect of feedback in the extra-classical RFs. Differently, Rao & Ballard have studied the contextual effects of the feedback connection in the classical RFs. In the subsection "*SDPC extends Rao & Ballard's PC model*" of the section "*Discussion*" (see lines 495-497), we have highlighted the strong similarities between the Rao & Ballard results (i.e. the end-stopping effects) and our results when the analysis is reduced to the classical RF (designated by a black square in Fig. 4 and Fig. 6). We have made more explicit the fact that our analysis is spatially extending the Rao & Ballard one.
- **3. About the missing definition of FISTA**. The reviewer #3 raised the fact that we used the FISTA acronym (Fast Iterative Soft Thresholding Algorithm) without previously defining it. We corrected our manuscript to remove as much as possible this acronym (following a suggestion made by reviewer #4). Nevertheless, we explicitly use this acronym is in the section "*Method*" in which we have defined

it (see line 690). We thank the reviewer #3 for pointing this error that made the article not easily understandable.

- **4. Clarification about the ℓ_0 pseudo-norm.** The ℓ_0-norm has the particularity not to be absolutely homogeneous. Said differently, the ℓ_0-norm is just counting the number of strictly positive scalars, and does not depend on their amplitude. So mathematically speaking, $\forall \alpha \in \mathbb{R} \| \alpha \cdot v \|_0 \neq \alpha \cdot \|v\|_0$ (v being a vector). That is why the ℓ_0-operator is termed a pseudo-norm. We have included a small sentence in the paper (line 117-118) to clarify this point (without entering into mathematical details to keep the paper clear and straightforward).
- **5. Clarification of the notion of number of channels and number of features in Table. 1**. We have adopted the machine learning convention to describe the size of our dictionary. A dictionary of size $[n_f, n_c, w, h]$ could be interpreted as a collection of n_f features of size $n_c \times w \times h$. n_c is then the number of channels of the representation on which we apply the convolution. For example, in the case of the first layer dictionary, $n_c = 3$ for color images (each channel encoding for red, green and blue colors respectively). For a dictionary in deeper layers, the number of channels of the representation, is actually equal to the number of features of the previous layer dictionary. w and h described the width and the height of the features. We have updated the section "*Method*", subsection "*Network parameters*" (lines 757-763) to make clearer the convention we are using. Note that, while correcting this specific part of the manuscript, we have noticed a typo in the dimension of the dictionaries (that is minor, and has no impact on the results, see Table 1.). This typo is now corrected. We thank the reviewer #3 for raising the need for clarification.
- **6. About the Eq.10, and the symbol** j. We have updated the "*Method*" section to define the j symbol, which is simply the complex number such that $j^2 = -1$ (see Eq. 10). In addition, we fully agree with reviewer #3 that our previous formulation of Eq.10 was not clear at all. This equation was updated to remove any mathematical claims, but we decided to keep it as it helps the reader to interpret the V1 representation in the complex space. This interpretation makes the understanding of the concept of interaction easier and more straightforward. In particular, we have updated Eq.10 to remove the sign $\equiv$ that means "equivalent" and which is indeed ambiguous in this case. Note that we have thoroughly reformulated the subsection describing the notion of interaction map in the "*Method*" section (see lines 770-846). We have included an additional figure (see Fig. 14) that makes the computational derivation of the interaction maps easier to grasp.
- **7. About the $atan2$ function and the Eq.14**. We have indeed forgotten to define the $atan2$ function. $atan2$ is a variant of the arctangent operator that returns positive angle for counterclockwise angle and opposite for clockwise angle. This function is commonly used to retrieve, in an unambiguous way, the argument of any complex number provided that the imaginary and the real part of the number are known. We have updated section "*Method*" to better define the $atan2$ function (see line 832), and we thank reviewer #3 for pointing this oversight.

Response to reviewer #4:

- **1. Reviewer #4 suggested that the numerous acronyms used in the paper make it hard to read.** We agree with reviewer #4 that there is too many abbreviations and acronyms in our paper. We decided then to remove most of the abbreviation and to keep only few of them (i.e. Predictive Coding (PC), Sparse Coding(SC), Sparse Deep Predictive Coding (SDPC), Receptive Fields (RFs), Wilcoxon signed-rank test (WT)). For those abbreviations, we made sure to frequently remind their definitions.
- **2. Reviewer #4 suggested to add a reference when introducing the Eq.1.** We have added the reference suggested by the reviewer #4 in section "*Results*", subsection "*Brief description of the SDPC*" (line 114) in which we introduce the Eq 1 that describes the general mathematical problem we are solving.
- **3. About the apparent sub-optimality of having $k_{\text{FB}} \neq 1$.** We fully agree with Reviewer #4 that minimizing Eq.2 with a $k_{\text{FB}} \neq 1$ seems to be sub-optimal to approximate the mathematical problem described in Eq.1. This sub-optimality is actually reflecting in Fig. 9 B and Fig. 10 B, that show a slightly degraded reconstruction performance when the input is not corrupted by noise and when the feedback strength is increased. Interestingly, increasing the feedback strength gives substantial advantages in terms of denoising ability compared to a SDPC trained with $k_{\text{FB}} = 1$. We have included a sentence in

the subsection "*Brief description of the SDPC*" (line 151-156) that highlights this interesting observation made by Reviewer #4.

- **4. About the bio-plausibility of the training of the SDPC**. We totally agree with the reviewer #4 that feedback connections in the brain are not the transpose of the feedforward connection. In our sentence line 86, we just wanted to highlight that in contrast to most of the unsupervised large scale models (like autoencoders), our model is adopting a local learning rule that is interpretable in terms of Hebbian learning. On our opinion, this is one of the interesting novelty lying in the SDPC. As our initial formulation was not clear, we reformulate to dissipate any misunderstanding (see section "*Introduction*" - paragraph §5 - lines 86-90).
- **5. About averaging the gradient across space.** We are not sure to well understand this specific comment made by reviewer #4. To the best of our knowledge, convolutional weight update does not have to be averaged across space dimensions (i.e. across the width and height dimension). Note that in our Eq. 4 (i.e. the Eq. 7 in the previous version), we abuse purposely the mathematical notations to make them more concise and clearer. Rigorously speaking, as we are optimizing the dictionaries using a stochastic gradient descent on mini-batches of images, we should have averaged the loss function across the number of images (not across space). Please find below the rigorous formulation of our stochastic gradient descent :

$$\mathbf{D}_i^{t+1} = \mathbf{D}_i^t - \frac{\eta_{L_i}}{N} \sum_{k=1}^{N} \left(\frac{\partial \mathcal{L}_i({}^{(k)}\gamma_i^t)}{\partial \mathbf{D}_i} \right)$$

  In this notation, N is the number of images composing the mini-batch, and the notation ${}^{(k)}\gamma_i^t$ denotes the latent variable corresponding to the image k, obtained at layer i and at time step t. This notation is definitely more rigorous, but also implies the use of multiple indices to describe our latent representation. We therefore prefer the concise mathematical notation.
- **6. About the update step of the inference process (Eq. 3).** Reviewer #4 commented the fact that in Eq.3, we have omitted the ℓ_1 penalty term in the update rule of γ_i. We would like to bring the reviewer attention on the soft-thresholding operator that makes this equation a bit different from classical gradient descent. The role of this operator is to sparsify the representation, and then to include the ℓ_1 penalty in the update rule. This loss function is directly deriving from the Iterative Soft Thresholding Algorithm. Note that we have added a small sentence, in section "*Brief description of the SDPC*" to make sure readers are aware that the soft-thresholding operator in Eq. 3 is a direct consequence of the ℓ_1 penalty in Eq. 2 (see line 162-165).
- **7. Reviewer #4 suggested to move the update and learning rule equation at the beginning of the section "*Results*".** We fully agree that moving these crucial equations at the beginning of the article would help the understanding of the inference and learning procedure. We then move these equations, previously in the "*Method*" section to the beginning of the section "*Results*".
- **8. Reviewer #4 raised the fact that we mixed stability and fixed points in Eq. 6**. We corrected our mistake in the core text and in our mathematical equations. We thank the reviewer #4 for bringing this point to our attention.
- **9. Reviewer #4 commented about the back-projection mechanism**. The Reviewer #4 is absolutely right to state that the back-projection mechanism is used only for visualization. We have added a small sentence in the "*Method*" section (subsection "*Back-projection mechanism*", line 724-725) to dissipate the misunderstanding. In addition, we have corrected the typo in Eq.8 raised by the Reviewer #4. We thank the reviewer #4 for raising all these details that make the article clearer.
- **10. Reviewer #4 commented about Eq. 10, and more generally about the lack of clarity of our interaction map analysis**. Eq. 10 is only proposing to the reader another interpretation of the V1 representation (i.e. γ_1). With this equation, we suggest that γ_1, originally defined in $\mathbb{R}$ could also be interpreted in the $\mathbb{C}$ space. This interpretation is making the interaction map computation easier and more straightforward. We have thoroughly updated the "*Method*" section that described the interaction map analysis (see lines 771-846). To make the explanation easier to follow, we have decomposed it in 3 steps : Step 1 being the neighborhood extraction, Step 2 is a normalization process, and Step 3 is the computation of the resulting orientation and activities. The complex interpretation of γ_1 is particularly useful in the Step 3. It allows to derive the computation of the orientation and the activities using the notion of argument and modulus of a $\mathbb{C}$ number. In addition we have added a figure illustrating the

different steps to derive the interaction map (see Fig 14). This figures is showing all the 3 steps on a very simplified case (only 4 features, few level of neural activity...) but we think it would be helpful for the reader to grasp the notion of interaction. We would like to thank Reviewer #4 for pointing out the need for clarification of the computation of interaction map.

- **11. Reviewer #4 suggested to add the mathematical definition of γ_i^{eff} and $D_i^{\text{eff}^T}$ in the legend of Fig. 2**. This suggestion is indeed making easier the reading and the understanding of Fig. 2. We thus added the underlying mathematical equation in the legend of Fig. 2.
- **12. Reviewer #4 commented about the lack of comparison between our results on neurophysiological observation**. We have updated our figures to include the biological trends when it was possible to do a consistent comparison (see the black arrows in Fig. 7 and Fig. 8). Note that the comparison is only qualitative as neurophysiological results are really dependent of the stimuli, the tested animals and the experimental protocol. In addition, we have included a paragraph to detail the similarities observed between the SDPC results and the neurophysiological trends (see subsection "*Comparing SDPC with neurophysiology*" in the section "*Discussion*", lines 537-559).
- **13 & 14 : Reviewer #4 commented on the Fig.3**. The Fig.3 is showing the evolution of the sparsity when varying the feedback strength. Biologically speaking, it is complicated to isolate the effect of the feedback on the sparsity of the neural representation. The neurophysiological literature converge toward a highly stimuli-dependent effect of the feedback. We wanted to highlight this specific similarity with biology when mentioning the increase of variability when the feedback strength is going up. We have updated the subsection "*SDPC feedback recruits more neurons in the V1 model*" to better clarify this link with biology (see lines 252-255). Generally speaking, the Fig.3 serves as an introduction of the analysis we are conducting in the rest of the manuscript. Before understanding the effect of the feedback strength at the selectivity and organizational level, we first expose the proportion of neurons activated by the feedback connection.
- **15 : Reviewer #4 suggests that the link between denoising and object recognition should be pointed-out.** We have added a sentence at the end of the subsection "*Effect of sparsity on denoising*" (line 452-456) to point-out this interesting point. We thank reviewer #4 for suggesting this modification that makes our network more appealing for additional downstream tasks.

Please find attached to this letter the new manuscript as well as the changes from the previously submitted version in supplementary information (i.e. tracked-changes format). Note that we had to submit all figures in separate files to fulfill the formatting requirement of the journal. We believe that the modifications brought to the manuscript are responding to the insightful comments of Reviewer #3 and Reviewer #4.

Sincerely yours,

The authors, Victor Boutin, Angelo Franciosini, Frederic Chavane, Franck Ruffier and Laurent Perrinet
