# Supplementary material for: Sparse deep predictive coding captures contour integration capabilities of the early visual system
Source: PLoS Comput Biol. 2021 Jan 26;17(1):e1008629. doi: 10.1371/journal.pcbi.1008629 (PMC7864399; doi:10.1371/journal.pcbi.1008629)
Supplement: Attachment — Submitted filename: RebuttalLetter.pdf [file pcbi.1008629.s011.pdf]

December 8, 2020

Victor Boutin
Institute of Neuroscience of la Timone
27, boulevard Jean Moulin
13005 Marseille, France
Email: victor.boutin@univ-amu.fr

Dear reviewers,

We wish to thank both reviewers, because their remarks have allowed us to greatly improve the clarity of the manuscript. The enclosed manuscript has been amended to answer the comments made by the reviewers. Note that all line references in this rebuttal letter correspond to line numbers in the tracked changes version of the article. Please find below a point-by-point description of the changes we have made in the manuscript:

Response to reviewer #4:

- **1. Reviewer #4 suggested to mathematically define the notion of effective dictionary earlier in the article**. We move the equation defining the notion of effective dictionary earlier in the article, i.e. in the section "*Brief description of the SDPC*" (see lines 138-140).
- **2. About the convolutional operator**. We would like to emphasize the fact that in the entire paper we use the 2D spatial convolution, as commonly adopted in the machine learning field. To make thinks clearer, let us do the math. Let's consider a dictionary $\boldsymbol{D}$ of size $[n_f, n_c, w, h]$, and an activity map $\boldsymbol{\gamma}$ of size $[n_f, w_m, h_m]$. Each element of the convolutional operation : $\boldsymbol{\gamma}_{i-1} = \boldsymbol{D}_i^T * \boldsymbol{\gamma}_i$ could be defined as in Eq. 1:

$$\begin{aligned} &\boldsymbol{\gamma}_{i-1} = \boldsymbol{D}_i^T * \boldsymbol{\gamma}_i \\ &\text{with} \quad \boldsymbol{\gamma}_{i-1}[j,k,l] = \sum_{m=1}^{n_c} \sum_{p=1}^{w} \sum_{q=1}^{h} \boldsymbol{D}_i^T[j,m,p,q] \times \boldsymbol{\gamma}_{i-1}[m,k-p,l-q] \\ &\text{s.t.} \quad k-p \in [\![1, w_m]\!] \quad \text{and} \quad l-q \in [\![1, h_m]\!] \end{aligned} \tag{1}$$

  Because this notation is too detailed to be straightforward for the reader, we just replace this element wise operation by a matrix-vector one as follow:

$$\boldsymbol{\gamma}_{i-1} = \mathcal{T}(\boldsymbol{D}^T) \times \boldsymbol{\gamma}_i = \boldsymbol{D}^T * \boldsymbol{\gamma}_i \quad s.t. \quad \mathcal{T} \text{ is the transformation of } \boldsymbol{D} \text{ into a Toeplitz matrix} \tag{2}$$

  In Eq. 2, the sign $\times$ denotes the matrix-vector multiplication. To facilitate the reading of the mathematical equation, we have purposely abuse the notation in the paper such that : $\boldsymbol{\gamma}_{i-1} = \boldsymbol{D}^T \boldsymbol{\gamma}_i = \mathcal{T}(\boldsymbol{D}^T) \times \boldsymbol{\gamma}_i = \boldsymbol{D}^T * \boldsymbol{\gamma}_i$. We agree that this abuse of notation is hiding all the computation behind the discrete 2D spatial convolution, and especially it is making implicit the position invariance of features (as the reviewer mentioned in the response). Nevertheless, we think this notation is making the paper clearer. In addition, it transforms the gradient operation to a simple matrix transposition (as shown in Eq. 3).

$$\frac{\partial \boldsymbol{D}^T \boldsymbol{\gamma}_i}{\partial \boldsymbol{\gamma}} = \boldsymbol{D} \boldsymbol{\gamma}_i \tag{3}$$

Consequently, this notation lightens our mathematical notation. To make this notation more explicit to the reader, we have added a subsection in the section "*Models and method*" (see lines 713-731) to better explain our notation.

- **3.a. About the location in the article of the interaction map explanation**. The reviewer #4 raised the fact that most of the explanations concerning the interaction map were located at the end of the article (section "*Models and method*") and thus does not match the reading flow. We have reorganized the article to answer this concern. We have created a subsection "*Interaction map to visualize the neural organization*", located in the main text of the article to detail and explain how the interaction are computed. We thanks the reviewer #4 for this comment that improves the overall clarity of the article (see line 253).
- **3.b. About the dimensionality of the activity map**. The reviewer #4 was concerned about the fact that the information of the dimensionality of the activity map was not explicitly detailed. To answer this concern we have added few sentences to clarified this specific point (see line 269-271, but also 124-128).
- **3.c. About the Equation 7 (i.e. Equation 10 in the previous version)**. We totally agree that this equation is confusing. We have introduced a new notation ($\gamma^{\mathbb{C}}$ that allows us to rigorously reformulate this equation to make it clearer. The new equation is then (see line 287) :

$$\forall \theta \in \{\theta_k\}_{k=0}^{n_f}, \forall x \in [\![1, w_m]\!], \forall y \in [\![1, h_m]\!], \quad \boldsymbol{\gamma}_1^{\mathbb{C}}[\theta, x, y] = \boldsymbol{\gamma}_1[\,\theta, x, y]\, e^{j\theta}$$
$$\text{s.t.} \quad j \in \mathbb{C} \quad \text{and} \quad j^2 = -1 \tag{4}$$

- **3.d. About the estimation of the θ parameter**. We think that both the reformulation of Eq. 6 and the clarification of the dimensionality of the activity map, should help both the readers and the reviewer to better understand how we computed θ. In a nutshell, θ corresponds to the features space of the activity map. Said differently, our activity map could be seen as a collection of 2D maps (retinotopically organized), in which each map is encoding for one specific feature. We have shown in Fig. 2.b and f, that the features of the first layer of our model were mostly oriented edges... Then by fitting Gabor filters with these edges, we can extract, for each feature, an angle that describes its orientation. In addition to the modification made for the responses 3.a and 3.b, we have updated the corresponding paragraph to make this idea clearer (see line 280-287).
- **3.e. About the neighborhood extract around the 10 most strongly activated neurons**. We agree with the Reviewer #4 that this could lead to a misunderstanding of our analysis. In the step one of the interaction map analysis, we are extracting a neighborhood for each of the 10 most strongly activated neurons (for a given preferred orientation). Consequently, we are generating 10 different cropped versions of γ_1 that are centered on the 10 most responsive neurons. We have reformulated the step 1 (see line 289-304), to make this point clearer.

Additional responses to comments from Reviewer #4:

- **1. Reviewer 4 suggested to better explain the structure of the activity map, as well as its computation before the section dealing with the interaction maps.** We agree with reviewer #4 that the dimensionality and the computation of the activity map is helpful for the understanding of the notion of interaction map. We then move the mathematical definition of the interaction at the beginning of the article (see lines 136-138). In addition, we have reformulated the paragraph before the one that is introducing the interaction map in order to more clearly map the link with the map dimensionality. As suggested by the reviewer #4, we have update the equation 7 to explicitly mention that θ x and y are discrete variables.
- **2. Reviewer 4 suggested to better explain what we meant by local plasticity.** Following the discussion with the reviewer #4 we have understood the plasticity rule of the SDPC is not local. We then have removed the 2 sentences in the section "*SDPC learns cortex-like RFs while performing neuro-plausible computation*" that stated the locality of the plasticity.
- **3. Reviewer 4 suggested to discuss the bio plausibility of the convolution**. We have added a small paragraph in the section to discuss the bio-plausibility of the convolutional operation (see lines 563-571)

Response to reviewer #3:

- **1. About the recent article : 'Predictive Coding approximated Backprop along abitraty computation graphs'**. We have cited this article in the introduction (see lines 75-77 of the article). Note that even if the mentioned article is indeed leveraging predictive coding framework, it is doing it in an discriminative way (i.e. for a classification purpose) whereas the framework we propose in this article is a generative model, i.e. made for reconstruction (in line with the original predictive coding framework proposed by Rao & Ballard).
- **2. About the definition of the ℓ_0-norm**. Following the suggestion of Reviewer #3, we have added a sentence (see line 115-116) to include the definition of the ℓ_0 pseudo-norm.

Please find attached to this letter the new manuscript as well as the changes from the previously submitted version in supplementary information (i.e. tracked-changes format). Note that we had to submit all figures in separate files to fulfill the formatting requirement of the journal. We believe that the modifications brought to the manuscript are responding to the insightful comments of Reviewer #3 and Reviewer #4.
Sincerely yours,

The authors, Victor Boutin, Angelo Franciosini, Frederic Chavane, Franck Ruffier and Laurent Perrinet
